# Loose Anagen Hair Associated with Wooly Hair Caused by a Heterozygous, Intronic *KRT71* Variant

**DOI:** 10.3390/genes16040459

**Published:** 2025-04-17

**Authors:** Elizabeth Phillippi, Marcelo Melo, Kelly N. Messingham, Hatem El-Shanti

**Affiliations:** 1 Department of Pediatrics, Carver College of Medicine, University of Iowa, Iowa City, IA 52242, USA; elizabeth-phillippi@uiowa.edu (E.P.); marcelo-melo@uiowa.edu (M.M.); 2 Department of Dermatology, Carver College of Medicine, University of Iowa, Iowa City, IA 52242, USA; kelly-messingham@uiowa.edu

**Keywords:** loose anagen hair syndrome, wooly hair, minigene, Keratin71, keratin intermediate filament, *KRT71*

## Abstract

Background: Loose anagen hair syndrome is a recently described genetic form of non-scarring alopecia that occurs in children and is due to poorly anchored hair shafts during the anagen phase. It can occur alone or in association with hair pathology or complex systemic phenotypes. Methods: We report a mother and daughter with loose anagen hair syndrome that is associated with wooly hair, although it shows variable expressivity. We studied the family using genomic sequencing and identified an intronic variant in their *KRT71* that segregates in an autosomal dominant pattern and is suspected to affect splicing in the tail domain of this hair follicle keratin. We studied this variant with a minigene experimental approach. Results: We provide experimental evidence that the identified intronic variant affects splicing in the tail domain, which is critical to the biomechanical properties of the keratin intermediate filaments. We demonstrate that it affects splicing by adding 12 bases to the mature transcript and consequently four amino acids to the peptide. Conclusion: We suspect that this variant is responsible for the poorly anchored and finely curled hair in the mother and daughter, which leads to a proposed diagnosis of autosomal dominant wooly hair, as well as loose anagen hair syndrome. We thus expand the variant spectrum of *KRT71* and its associated phenotypes to include both disorders.

## 1. Introduction

Loose anagen hair syndrome (LAHS) is a rare genetic form of non-scarring alopecia that is due to poorly anchored hair shafts in their follicles during the growth (anagen) phase [1,2]. It is associated with hair thinning that is usually diffuse but can be patchy; the anagen hairs can be easily and painlessly extracted before they complete their growth cycle, leading to reduced hair length [3,4]. LAHS can occur alone, may be part of a complex phenotype, such as Noonan [5] and tricho-rhino-phalangeal [6] syndromes, or may accompany other hair shaft pathologies, such as wooly hair or unmanageable hair [7,8], thus exhibiting phenotypic heterogeneity. LAHS occurs sporadically, but can be familial, mostly following an autosomal dominant form of inheritance [9]. It is suggested that premature or defective keratinization occurs in the inner root sheath cells, which decreases their ability to hold on to the growing hair shaft [2].

The hair loss disorders that occur in the pediatric age group and that are associated with bullying, decreased self-esteem, and reduced quality of life include LAHS and another overlapping condition, short anagen hair syndrome (SAHS) [10,11]. About half of LAHS and SAHS patients and their caregivers report negative psychologic symptoms in the form of anxiety, depression, low self-esteem, and body dysmorphia and about a third report bullying or mistreatment by their peers [11]. The psychological impact of LAHS and SAHS is comparable to the impact of alopecia areata on children [10,11].

An earlier study suggested that LAHS is a keratin disorder, and identified a pathogenic variant in *KRT75* in three out of the nine families that were studied [9]; however, this has not been replicated. Keratin75 is expressed in the companion layer, which was previously considered the innermost layer of the outer root sheath (ORS). A recent study identified biallelic missense variants in *TKFC* in hypotrichosis with loose anagen hairs, presenting functional experimental evidence to the pathogenicity of the two variants [12]. These biallelic variants were found in one affected individual in 1 out of 15 studied families and biallelic variants in *TKFC* were implicated in other phenotypes that do not include hair abnormalities [13]. The microscopy of hairs extracted from LAHS patients shows absent inner and outer root sheaths, misshapen bulbs, and ruffled or peeling cuticles proximal to the root [2,4]. Additionally, thin sections of scalp tissue containing affected hair follicles show disorganized keratin fibers, enlarged or distorted cells of the inner root sheath (IRS) layers, and edema between the anchoring layers of the Henle and Huxley cells, which help to bind normal hair shafts to the follicle [14].

Autosomal dominant wooly hair (ADWH) is characterized by fine, tightly curled, and short hair shafts, and is due to pathogenic variants in *KRT74* [15] [OMIM 194300] and *KRT71* [16] [OMIM 615896]. The association of wooly hair with LAHS has been previously described [17,18]; however, a genetic study was not conducted in these case reports. Keratin71 is expressed in all three layers of the IRS and is a component of the intermediate filaments in IRS cells [19].

We describe a 16-year-old girl diagnosed with LAHS and reported in the LAHS literature [3], and her affected mother, who has a milder phenotype. They both have an intronic variant in *KRT71* that can alter its splicing, resulting in four additional amino acids in the tail domain of the protein. In addition to the LAHS phenotype, both have a hair texture that fits the wooly hair description, suggesting that heterozygous variants in *KRT71* can be responsible for ADWH associated with LAHS, which is a rare association [17,18].

## 2. Materials and Methods

### 2.1. Clinical Information

We obtained a release of information to acquire the medical records from the Children’s Hospital of Philadelphia, where the proband was treated since she was 5 years of age (2013).

### 2.2. Biologic Samples

We obtained saliva samples (OGR-600, DNAgenotek, Inc., Ontario, Canada) for DNA extraction from all participating family members, after obtaining informed consents from the participants or their authorized legal representative. We obtained shed hairs from both the proband and her mother. The root ends of hairs were tape-mounted and examined with a dissecting microscope at 10–63× magnification using direct illumination. We reviewed the proband’s medical records and obtained a detailed family history.

### 2.3. Next-Generation Sequencing (NGS) and Variant Annotation

Three hundred cycles of massively parallel genome sequencing (GS) were performed using the NovaSeq6000 platform (Illumina^®^, San Diego, CA, USA) and KAPA Hyper Prep Kit (Roche, Indianapolis, IN, USA) at the Iowa Institute of Human Genetics (Iowa City, IA, USA). The paired-end reads were analyzed using the DRAGEN platform for read alignment and variant calling (Illumina^®^, San Diego, CA, USA). Variants were annotated using the VarSeq platform (Golden Helix, Bozeman, MT, USA). These variants were filtered using the following parameters: quality score ≥ 25, read depth ≥ 8x, alternative allele frequency ≤ 0.0005 or missing from UK10K (https://www.uk10k.org/), 1000 genomes/1 KG (https://www.internationalgenome.org/) [20], gnomAD v.2.1 (https://gnomad.broadinstitute.org/), and Kaviar (https://db.systemsbiology.net/kaviar/, accessed on 14 April 2025) [21]. Filtered variants were further considered if their CADD score was ≥15 [22] or SpliceAI calculated a delta score for any category ≥ 0.2 [23]. Appropriately rare and potentially pathogenic variants in the proband were then checked for segregation in her mother and absence in the unaffected brother. Variants in the father were genotyped by Sanger sequencing, since he decided to participate in the study after the genome sequencing was completed. The variants were then filtered biologically based on their role in hair follicle biology and hair shaft structure, hair follicle morphogenesis and cornification, signaling pathways for hair cycling, mechanotransduction, and extracellular matrix integrity and remodeling.

### 2.4. Minigene Assay

**Figure 2:**
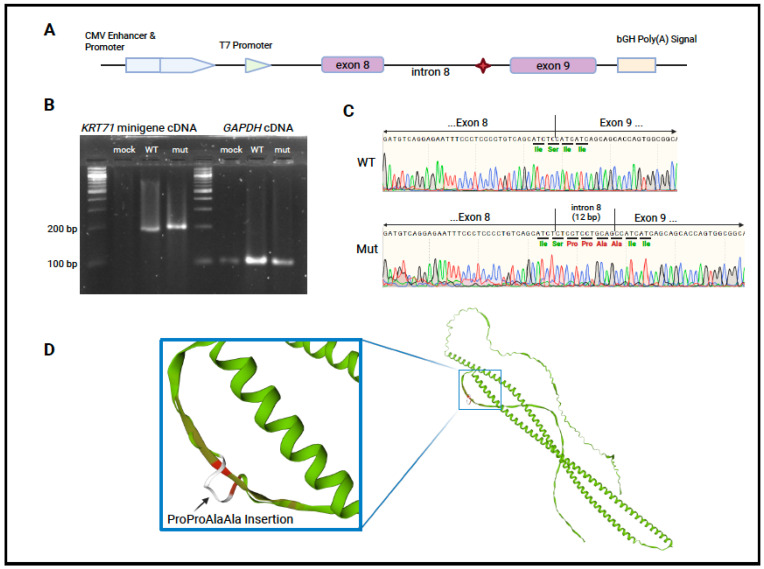
(**A**) Schematic of the minigene design. The intronic variant is indicated with a red diamond. (**B**) Agarose gel of cDNA products using total RNA as a template. RT-PCR of RNA from all transfections produced a GAPDH band of a size confirming the presence of mRNA template from each condition. No minigene products were detected in the mock transfection, and bands of a different size were detected in the cells transfected with the WT and mutant vector. (**C**) Sanger results of minigene cDNA products sequenced with the same primers that were used to amplify them. The mutant sequence includes 12 bp from the intron that are not present in the natively spliced product. (**D**) Predicted tertiary structure based on amino acid sequence generated with the SWISS model workspace. The mutant and reference predicted structures are overlaid and colored green where the models agree. The inset highlights the introduced residues of the tail domain, which add a small loop structure in this region which can be seen in white overlaying the reference structure which is colored from olive to red where the models deviate, with red denoting more extreme deviation. Created with BioRender.com.

The minigene assay was adapted from a previously reported method [24] to test the effect of the intron 8 variant, shared by the proband and her mother, on *KRT71* splicing. Briefly, pcDNA3.1+ (Genscript, Piscatawny, NJ, USA) was linearized with EcoRI-HF and XbaI (New England Biolabs, Ipswich, MA, USA). The backbone was gel purified (QIAquick^®^ Gel Extraction Kit, Qiagen, Germantown, MD, USA), and a 1065 bp insert consisting of *KRT71* (NM_033448.3) exon 8 (35 bp), intron 8 (821 bp), and exon 9 (212 bp) with 25 bp overlaps matching the linearized plasmid was assembled from gBlocks (IDT, Coralville, IA, USA) and cloned into the vector with the Gibson Assembly Cloning Kit (New England Biolabs, Ipswich, MA, USA) as seen later in Figure 2A. The gBlock containing intron 8 was synthesized with either the reference sequence or with the patient variant: (G > A) 14 bp upstream of exon 9. Successfully transformed colonies were screened with Sanger sequencing to confirm the completeness and correctness of the insert sequence and both a reference and mutant construct were confirmed. A normal clone, a mutant clone, or sterile PBS was transiently transfected into HEK293 cells, which do not natively express *KRT71.* The cells were seeded in 1 mL of media in a 12-well culture plate at a concentration of 1 × 10^5^ cells. Cells were allowed to adhere overnight in a 37 °C incubator with 5% CO_2_ and were transiently transfected with 1.6 µg of vector using Lipofectamine™ 2000 (Invitrogen™, Carlsbad, CA, USA), following the manufacturer’s recommendations. Total RNA was extracted after 24 h using TRIzol™ reagent (Invitrogen™) and PureLink™ RNA Mini Kit (Invitrogen™) and then treated with DNase I (PureLink™ DNase Set, Invitrogen™) on the column before final elution. RT-PCR was performed using the ZymoScript™ One-Step RT-qPCR Kit (Zymo Research, Irvine, CA, USA), either *KRT71* exon 8- and exon 9-specific primers or *GAPDH*-specific primers, and 5 µL of total RNA. The cDNA products were resolved on a 1% agarose gel and sequenced to identify the splice products. All transfections and extractions were performed in triplicate for each construct and a mock transfection (PBS) was used as a negative control.

### 2.5. Sanger Sequencing

The cDNA products from the minigene experiment were submitted to the Iowa Institute of Human Genetics for Sanger sequencing. The target cDNA was amplified using the same primers used during RT-PCR and amplified sequences were fluorescently labeled using BigDye Terminator v3.1^®^ (Applied Biosystems, Waltham, MA, USA). Chromatograms were interpreted and aligned to a reference sequence using SnapGene v7.2 (San Diego, CA, USA).

## 3. Results

### 3.1. Clinical History and Phenotype

**Figure 1:**
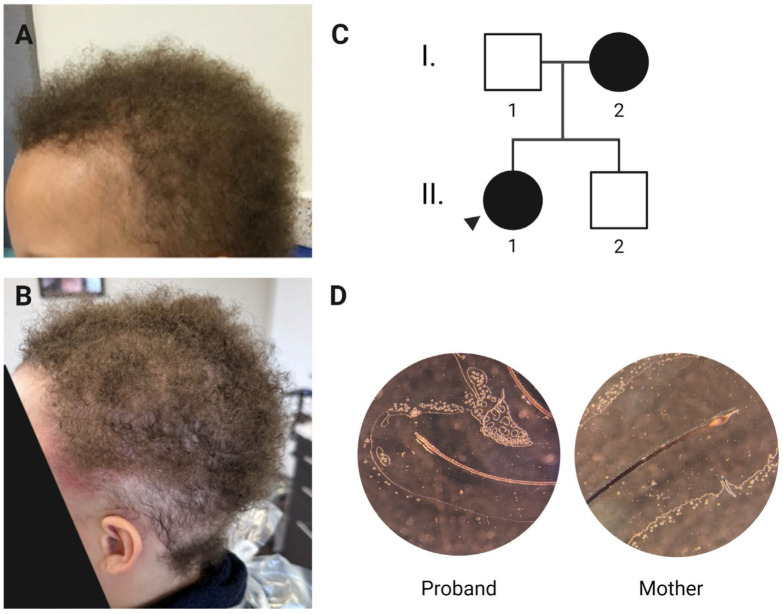
(**A**) Proband’s hair at age 6 (**B**) and age 16. The reddened patches on her temples are a reaction to the glue used for her wig and the hair near her nape is intentionally shaved. (**C**) Pedigree of the family suggesting an autosomal dominant pattern of inheritance. (**D**) Hairs from mother and proband under 50X magnification, tape mount. The proband’s hair lacks root sheath and is in anagen phase with tapered, dysmorphic root. The mother’s hair is normal telogen-phase club hair. There is no curl present in the mother’s hair owing to chemical treatment for cosmetic straightening. Created with BioRender.com.

The proband, currently a 16-year-old girl, has fine, short, tightly curled hair that seemingly does not grow. Her hair is anchored poorly to her scalp and can be dislodged with a light touch. She was bald at birth, and by two years of age, she had developed “peach fuzz” hair. By the age of six years, her hair was approximately two inches long and appeared to be growing very slowly (Figure 1A). Her hair is uniformly sparse but more so at the temples. She is reported in the medical literature as having LAHS after a positive pull test was conducted [3]. On examination of her medical records from the Children’s Hospital of Philadelphia, she has a positive hair-pull test in which most of the pulled hairs were in the anagen phase, lacking the inner and outer root sheaths and showing ruffled cuticles. The clinical diagnoses of SAHS and wooly hair syndrome were introduced in her medical records around puberty. Despite expectations that both her hair growth pattern and its easy pluckability would improve with age, both persisted; currently, she partially shaves her hair to allow the adhesion of a wig (Figure 1B). Her eyebrows, nails, teeth, and sweating are all normal.

The proband’s mother describes a similar course as her daughter, although her symptoms improved with the onset of puberty. Her hair grows much more slowly than average (~1 inch per year) and remains sparse at her temples, where she uses a topical minoxidil to induce vellus hair growth. She chemically straightens her hair, leaving her hair samples lacking the characteristic tight curls of wooly hair.

More than 50% of the shed hairs from the proband were loose anagen hairs (13/25) that showed a lack of inner and outer root sheaths and ruffled cuticles, while shed hairs from her mother were mainly in the telogen phase (10/11) (Figure 1D). Normal shedding typically consists of telogen hairs, further demonstrating the improvement in the mother’s LAHS phenotype.

### 3.2. Variant Identification and Analysis

We performed short-read GS on the proband, her mother, and her unaffected brother and annotated and filtered the variants as described in the Materials and Methods Section. Six heterozygous missense variants in *PIEZO1*, *CUX1*, *COL5A1*, *MMP2*, *CHD9*, and *TCF3* were identified, as well as two heterozygous possible splice-site variants in *KRT71* and *MMP8*. Heterozygous variants in *PIEZO1* are described in dehydrated hereditary stomatocytosis with or without pseudohyperkalemia (OMIM 194380) [25,26]. Heterozygous variants in *CUX1* are associated with global developmental delay with or without intellectual impairment (OMIM 618330) [27,28]. Heterozygous variants in *COL5A1* are associated with Ehlers–Danlos syndrome, classic type 1 (OMIM 130000) [29], and multifocal fibromuscular dysplasia (OMIM 619329) [30]. Heterozygous variants in *TCF3* are associated with agammaglobulinemia 8A (OMIM 61694) [31]. *MMP2* is associated with an autosomal recessive disorder and *CHD9* and *MMP8* have not been associated with a genetic phenotype. No variants were detected in *KRT75* or *KRT74*.

Both the proband and her mother had a heterozygous G > A variant in *KRT71* (NM_033448.3:c.1361-14G > A) (rs367619481), which was not found in her unaffected brother or her father (by targeted Sanger sequencing). Pathogenic variants in *KRT71* have previously been reported to be responsible for ADWH with hypotrichosis [16], but not LAHS. SpliceAI (Broad Institute) showed moderate in silico support for the loss of the canonical acceptor site (Δ score = 0.46) and weak support for a new acceptor site 2 bp downstream from the variant site (Δ score = 0.14). We performed a minigene assay to test this predicted loss of a canonical acceptor site, and the potential gain of a new acceptor site.

The variant creates a splice site 12 bp upstream of the canonical acceptor splice site of exon 9, thus adding 12 base pairs to the mature mRNA and four amino acids to the translated protein (Figure 2B–D). This causes the in-frame insertion of Pro-Pro-Ala-Ala between the reference exons. The 523-amino-acid reference sequence for KRT71 and the new 527-amino-acid mutant sequence were submitted to the SWISS model workspace [32] and the resulting structures were overlaid to assess their similarity; the new amino acids were predicted to add a small loop structure to the tail domain, with the preceding domains an having unaltered predicted geometry (Figure 2D).

## 4. Discussion

Keratin intermediate filaments (KIFs) are formed of heterodimers of type I and type II keratins which achieve longitudinal growth through the head–tail interaction of anti-parallel tetramers [33]. Keratin71 is a critical component of the hair follicle and is found in all three layers of the IRS [19]. The variant in the helix initiation motif of *KRT71* in the ADWH cases leads to the collapse of the KIF network owing to its impaired ability to appropriately heterodimerize with its type I partners [16]. The *KRT71* variant reported here is located between exons 8 and 9 which, combined, comprise the tail domain of human *KRT71* (Figure 2A) [33].

While KIF filaments can grow and assemble when missing a tail domain, KIFs formed from these truncated monomers do not retain the viscoelastic properties of networks formed from whole keratins and exhibit significantly less strain stiffening when subject to mechanical force [34]. Keratin network collapse, as well as the altered expression of cell adhesion junctions (*DSG1*) and the downregulation of MAPK signaling, was observed in an in vitro model of a missense variant in the tail domain of a type II keratin (*KRT5)* [35]. We predict that the alteration of the tail domain of *KRT71* seen in our patients disturbs the formation of the KIF in their IRS cells. This disruption may deform the cells of this layer of the follicle and allow them to be more easily dislodged from the hair shaft with the application of relatively low shear force. The disruption or collapse of KIF and the resulting loss of the underlying structure could explain the malformed and torn cells of the IRS seen in LAHS microscopy in the literature [14].

Homozygous variants in *Krt71* are responsible for curly pelage in cattle (*Krt71*: c.281delTGTGCCCA;p.Met94AsnfsX14) [36,37], dogs (*Krt71*:c.1266_1273delinsACA; NP_001183958.1:p.Ser422ArgfsTer212 and *Krt71*:c.451C>T;p.Arg151Trp) [38,39], and cats (*Krt71*:c.445-1G>C; p.149-154 del6-amino acid from exon 2 and *Krt71*:c.816+1G>A; early TER 27 bp after alternate splice) [40,41]. Homozygous and bi-allelic *Krt71* variants in Sphynx cats result in a naked feline because the hairs of the coat form but lack the appropriate hair bulb morphology to anchor properly, resulting in truncated hairs and continuous shedding [41]. The homozygous frameshift variant that causes curled hairs in Hereford cattle also results in poorly anchored, curly hairs which are easily plucked [36]. The negative social repercussions of hair loss in cattle is highlighted in one study where one of the affected calves had to be isolated because other calves treated it with aggression [37].

Despite the implication of *KRT71* variants in ADWH [16], to our knowledge this is the first report of *KRT71* variants being responsible for the LAHS phenotype in association with ADWH. This case report expands the phenotype, as well as the spectrum, of pathogenic *KRT71* variants. The diagnosis of the proband in this family and her mother should encompass both LAHS and ADWH due to the observed fine, tight curly hair, as well as the painless and easy pluckable anagen hairs that show the typical loose anagen hair phenotype. The phenotype exhibited here is like the previously reported phenotype [17], especially as it pertains to the thinning observed in the frontoparietal regions in both the proband and her mother.

The *KRT71* variant identified in this report exhibits variable expressivity, where the LAHS phenotype improved with the onset of puberty in the mother but not in her daughter; however, the tight curls of the ADWH did not change for both. This variability may be attributed to other genetic factors or environmental factors and may also be attributed to the proportion of the spliced-out mRNA product. It is notable that the daughter resorted to wearing a long-term wig to avoid mistreatment by her peers, and that her mother straightens her hair for socially cosmetic reasons. It would be advisable to test for *KRT71* variants in other patients to verify the association between *KRT71* variants and LAHS.

## 5. Conclusions

In this case report, we demonstrate that the loosely anchored anagen hair phenotype and the tightly curled wooly hair phenotype may co-occur in the same patient secondary to disease-causing variants in *KRT71*, which is classically reported to cause only ADWH. We demonstrate that an intronic variant affects splicing and produces a peptide that is four amino acids longer in its tail domain, which we anticipate affects the secondary and tertiary structure of the mature protein and affects its interaction with its KIF partner.

## Data Availability

The original contributions presented in the study are included in the article, further inquiries can be directed to the corresponding author.

## Abbreviations

The following abbreviations are used in this manuscript:

LAHS	Loose Anagen Hair Syndrome
SAHS	Short Anagen Hair Syndrome
ORS	Outer Root Sheath
IRS	Inner Root Sheath
ADWH	Autosomal Dominant Wooly Hair
KIF	Keratin Intermediate Filament
